# Routine creatine kinase testing does not provide clinical utility in the emergency department for diagnosis of acute coronary syndromes

**DOI:** 10.1186/s12873-019-0251-4

**Published:** 2019-07-09

**Authors:** Evan J. Wiens, Jorden Arbour, Kristjan Thompson, Colette M. Seifer

**Affiliations:** 1 Department of Internal Medicine, Max Rady College of Medicine, University of Manitoba, Room GC430, Health Sciences Center, 820 Sherbrook Street, Winnipeg, Manitoba R3A 1R9 Canada; 2 Department of Emergency Medicine, University of Manitoba, Winnipeg, MB Canada; 3 Section of Cardiology, Department of Internal Medicine, University of Manitoba, Winnipeg, MB Canada

**Keywords:** Creatine kinase, Myocardial infarction, Diagnosis, Unnecessary testing

## Abstract

**Background:**

Despite the high sensitivity and negative predictive value of contemporary high-sensitivity troponin T assays (hsTnT), creatine kinase (CK) continues to be routinely tested for the diagnosis of acute coronary syndrome (ACS). We conducted a study to identify the clinical utility of routine CK measurement, its relevance in clinical decision making in the era of hsTnT, and the potential cost-savings achievable by limiting its use.

**Methods:**

We conducted a retrospective review of all adult patients presenting to a tertiary care center in the year 2017. We identified patients presenting with cardiac complaints who had non-diagnostic hsTnT and positive CK. These patients underwent chart review to determine whether a diagnosis of AMI was made.

**Results:**

A total of 36,251 presentations were reviewed. 9951 had cardiac complaints and 8150 had CK measured. 82% of these patients had hsTnT and CK measured; 2012 of these patients had non-diagnostic hsTnT with positive CK. Of these 2012 patients, only 1 was subsequently diagnosed with AMI (0.012%). CK provided no diagnostic benefit over hsTnT alone in > 99.9% of cases. With a cost for CK of $4/test, we estimated that routine CK testing costs at least $32,000 per year in our center, and over $100,000 per year across the region.

**Conclusion:**

Routine CK testing does not provide a significant benefit to patient care and therefore represents an unnecessary system cost. Routine CK testing for the diagnosis of AMI should be eliminated from emergency departments in the era of hsTnT assays.

## Background

Measurement of serum creatine kinase (CK) has long been considered a standard test in the work-up of chest pain in the emergency department (ED) and continues to be used routinely in institutions across North America [1]. However, with the advent of high-sensitivity cardiac troponin (hsTnT) assays, the clinical utility and cost-effectiveness of CK has come into question [1–5]. CK lacks tissue specificity and is elevated in numerous pathologic processes including muscle injury, trauma, and renal insufficiency, whereas detection of cardiac troponins in serum is extremely specific to cardiac injury [6]. Contemporary hsTnT assays have a sensitivity of 92–100% and a negative predictive value (NPV) of 96–100%, in part due to their increased concentration in cardiomyocytes compared to CK [7].

There is a paucity of data defining the clinical utility of CK in the age of current hsTnT assays. In one recent study, Volz et al. [2] reviewed a large cohort of approximately 11,000 patients in which troponin T and CK-MB were measured. Only 11 had positive CK-MB index and negative troponin T assays. Of these, none were discharged with the diagnosis of myocardial infarction and none died within the next 30 days. However, this study is limited by the small sample size of only 11 patients, and by the exclusion of patients with elevated hsTnT at baseline, such as those with chronic heart or renal failure.

Despite the widespread acceptance of hsTnT assays as the superior laboratory diagnostic modality for AMI [8], CK continues to be routinely measured alongside hsTnT in the workup of chest pain at many emergency departments. If CK provides no significant utility in the diagnosis of AMI, then it represents an unnecessary cost to the healthcare system for minimal benefit. We sought to examine the rates with which CK is currently being measured in a tertiary academic center, and its usefulness in clinical decision making for the diagnosis of AMI in a real-world contemporary clinical setting. Our second objective was to estimate the cost-effectiveness of utilizing CK for diagnosing AMI and to estimate the potential monetary savings that could be realized if its use was limited.

## Methods

We conducted a retrospective review of all patients aged 18 years and over presenting to the ED at a single tertiary care center between January 1, 2017 and December 31, 2017. Presentations were further screened by presenting complaint as categorized by the Canadian Emergency Department Information System (CEDIS). The study site is a large, urban hospital which serves as the regional cardiac referral site with cardiac catheterization laboratories, cardiac intensive care units, cardiology inpatient wards and a cardiac surgery program.

We identified patients with CEDIS complaints likely to prompt hsTnT and CK testing and also to represent all acute coronary syndromes; specifically, *Chest Pain* (with or without cardiac features), *Palpitations, Presyncope/Syncope, Shortness of Breath, Generalized Weakness, and Vertigo*. In addition, patients admitted from the ED with an admission diagnosis of non-ST-elevation ACS (NSTE-ACS) were reviewed regardless of CEDIS complaint to determine whether or not CK played a role in decision making. Patients were excluded if they were younger than 18 years of age at the time of presentation, had CEDIS complaints other than those listed above, were diagnosed with ST-elevation ACS (STE-ACS), or were transferred following a diagnosis of AMI made at another center.

At the study site, patients presenting with the relevant CEDIS complaints had bloodwork drawn at the time of triage including a CK and hsTnT; this is per a previously established regional protocol irrespective of the patient’s medical comorbidities or age. Physicians were also able to order testing for cardiac biomarkers after assessing a patient if this was not already done as per the protocol, and were able to order serial cardiac biomarkers at their discretion. The time interval for repeating cardiac biomarkers was variable and physician dependent, although was between two and four hours for the majority of patients. The assay kits for both CK and hsTnT at the study site laboratory are standard commercial assays manufactured and provided by Roche Canada.

Among unique patient presentations that met the inclusion criteria, we analyzed laboratory data from those visits. This laboratory analysis was done without knowledge of medical history, current medications, or ECG interpretation. We identified those patients who had both “non-diagnostic” hsTnT and “positive” CK. For the purposes of our study, we defined diagnostic hsTnT as values greater than the 99th percentile of a reference population (14 ng/L) and increasing by ≥5 ng/L after at least 1 h; this definition was previously shown to have high sensitivity (99.6%) and specificity (95.7%) for diagnosing AMI [9], and would generally prompt further testing and cardiology referral in our institution. Other hsTnT values, including those that were elevated but not trending upwards, were considered non-diagnostic. This was done to include patients with chronically elevated hsTnT, such as in patients with chronic kidney disease. A positive CK value was defined as any value above the upper limit of normal as defined by our institution’s laboratory (175 U/L for females, 190 U/L for males), or any increase within 24 h (even within the normal range). Serial decreases in hsTnT were not considered to be diagnostic of AMI for the purposes of our study, and patients with decreasing hsTnT and positive CK underwent detailed chart review. With this strategy, we aimed to ensure that any patient in which CK might have contributed to clinical decision making in the diagnosis of AMI was included.

Patients identified as having a non-diagnostic hsTnT assay and positive CK underwent a thorough electronic chart review to determine if a diagnosis of AMI was made by an attending cardiologist during the patient’s presentation. This was determined by the presence of AMI as a discharge or admission diagnosis, or by documentation in the patient’s chart of a diagnosis of AMI by the attending cardiologist or their designate. As a secondary outcome, we evaluated the 30-day incidence of major adverse cardiovascular events (MACE), defined as STE-ACS, NSTE-ACS, or need for urgent, unplanned percutaneous or surgical revascularization.

The data collection and chart reviews were done independently by two reviewers, and diagnoses were extracted from the patient chart as described above. For any cases in which uncertainty regarding the final diagnosis existed, consensus was reached between the two independent reviewers. If no consensus was reached, the chart was reviewed by a senior reviewer, who was blinded to CK levels, for determination of the final diagnosis.

This study was approved by the University Health Ethics Research Board as well as the local study site.

## Results

**Fig. 1:**
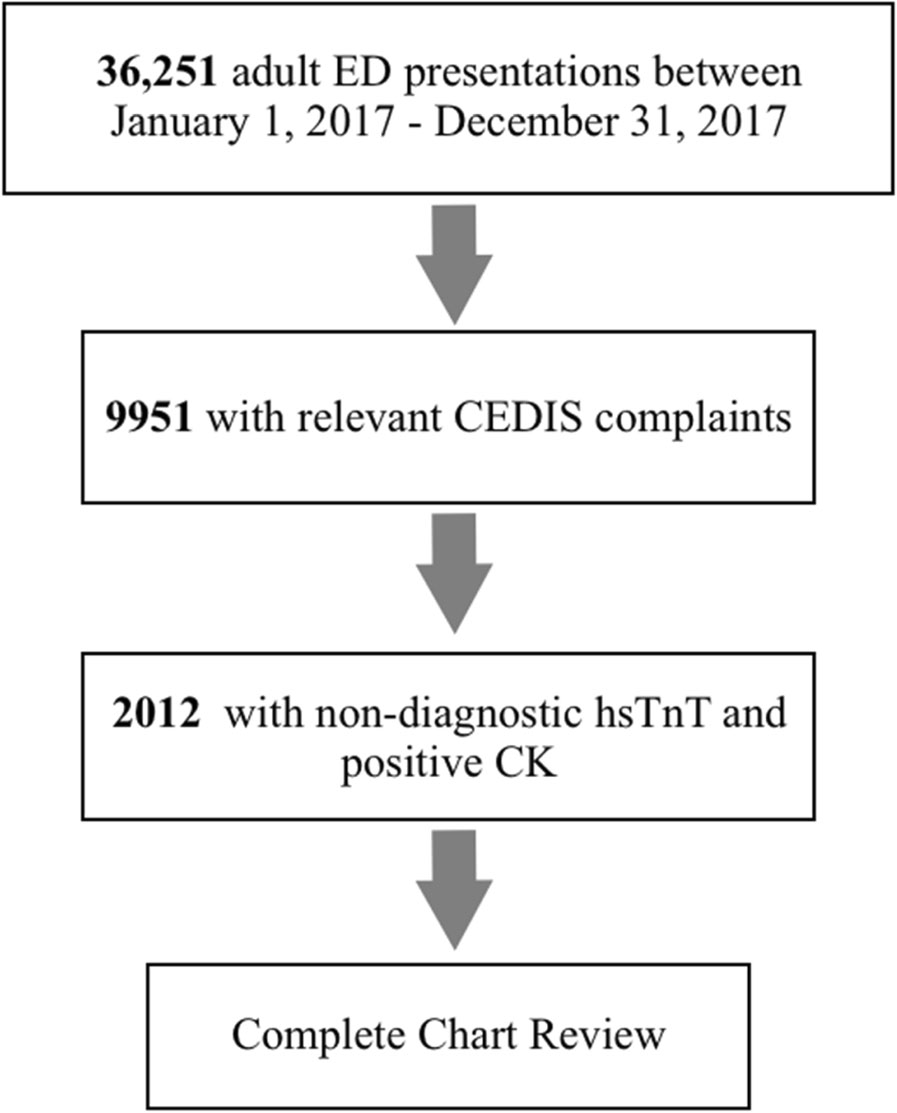
A total of 36,251 emergency department presentations were reviewed. Of these, 9951 had relevant CEDIS complaints. 2012 had non-diagnostic hsTnT and positive CK, prompting thorough chart review. *CEDIS; Canadian Emergency Department Information System, ED; emergency department, CK; creatine kinase, hsTnT; high-sensitivity troponin T*

A total of 36,251 ED presentations were reviewed; 9951 had relevant CEDIS complaints (Fig. 1), representing 27.5% of all ED presentations (Table 1). There was significant variation between the CEDIS categories with respect to the proportion of patients investigated for AMI by cardiac biomarker testing. Overall, 82.1 and 81.9% had hsTnT and CK measured, respectively (Table 1). The CEDIS complaint for which biomarkers were most frequently measured was *Chest Pain with Cardiac Features*, which prompted biomarker testing in > 97% of cases. Of the included complaints, biomarkers were measured least commonly in *Vertigo* (< 40%). Across all CEDIS categories, CK was ordered in conjunction with hsTnT in nearly all instances. 9524 of these patients (95.7%) were discharged home from the ED, and 427 (4.3%) were subsequently admitted to hospital.

**Table 1:** Out of a total of 9951 patients presenting with cardiac CEDIS complaints, 82.1 and 81.9% of patients, respectively, had hsTnT and CK measured. *Chest Pain (Cardiac Features)* prompted biomarker testing most frequently. 1.2% of patients presenting to the ED were subsequently admitted with diagnoses of NSTE-ACS. *hsTnT; high-sensitivity troponin T, CK; creatine kinase, NSTE-ACS; non-ST-elevation ACS*

	Total Patients	Prevalence (*n* = 36,251)	hsTnT Measured	CK Measured
Chest Pain	404	1.1%	96.3%	95.8%
Chest Pain (Cardiac Features)	3555	9.8%	97.6%	97.4%
Chest Pain (Non-cardiac Features)	1040	2.9%	67.5%	67.4%
Generalized Weakness	746	2.1%	62.6%	62.9%
Palpitations	1033	2.8%	88.2%	87.8%
Shortness of Breath	1600	4.4%	68.2%	68.2%
Syncope	709	2.0%	75.9%	75.7%
Vertigo	437	1.2%	39.6%	39.1%
Admitted with NSTE-ACS (any CEDIS complaint)	427	1.2%	100%	100%
Combined	9951	27.5%	82.1%	81.9%

We identified 2012 patients who had a non-diagnostic hsTnT and positive CK. Of these, only one patient was diagnosed with AMI and one additional patient was identified as having had a 30-day MACE (Table 2). In > 99.9% of patients, CK measurements did not contribute to clinical decision making in the diagnosis of AMI in the ED. Almost all of these 2012 patients were diagnosed with non-cardiac causes of chest pain. There were no patients in the cohort with a positive CK and serially decreasing hsTnT who were diagnosed with AMI.

**Table 2:** Of the 9951 patients presenting with cardiac CEDIS complaints, 2012 had negative hsTnT and positive CK. Of these, only one patient was subsequently judged to have had AMI, and one additional patient had a MACE within 30 days. *CK; creatine kinase, hsTnT; high-sensitivity troponin T, AMI; acute myocardial infarction, MACE; major adverse cardiac event*

Total patients	CK measured	hsTnT measured	-TnT/+CK	Diagnosis of AMI	30-day MACE
9951	8150	8167	2012	1	2
	81.9%	82.1%	20.2%	0.012%	0.025%

The cost of a single CK assay at our institution is approximately $4/test. For the 8150 patients in which CK was measured, this roughly equates to a cost of at least $32,000 per year at our institution. Across our municipal health region, ~ 25,000 patients per year present to hospital with cardiac complaints prompting cardiac biomarker testing. CK testing therefore is estimated to represent a cost of at least $100,000 per year in the region.

## Discussion

Measurement of CK in the emergency department in the diagnosis of AMI continues despite widespread acceptance of the superiority of hsTnT assays [1]. This retrospective review examined patient presentations to a tertiary emergency department over a one year period and showed that CK did not provide any diagnostic utility in > 99.9% of patients. In addition, it was estimated that CK accounted for >$32,000 per year of unnecessary cost in the study center and > $100,000 per year across the regional health regional. Our findings are consistent with those of Volz et al. [2] and other studies suggesting a limited role for CK [1–5].

There were many patients in this retrospective cohort that were diagnosed with AMI, and none had a truly negative hsTnT and positive CK. One patient was diagnosed with AMI with a positive CK and non-diagnostic hsTnT by our study definition (representing 0.012% of the cohort); this patient’s hsTnT was elevated but non-trending. In addition, it is unclear whether this case was truly an AMI. The patient presented with atypical chest pain and minimally elevated and non-trending hsTnT in the setting of congestive heart failure. The patient underwent coronary angiography, which did demonstrate obstructive coronary artery disease. However, despite placement of drug-eluting stents to the obstructive lesions, the patient’s chest pain did not resolve and the diagnosis remained in question. To err on the side of caution, we counted this case as the only AMI in our study.

Our definition of non-diagnostic hsTnT was conservative, and our definition of positive CK was very inclusive. We thereby sought to give CK every possible opportunity to show clinical utility. Despite this, CK was found to provide no benefit regarding clinical decision making for diagnosing AMI in > 99.9% of cases. These findings further affirm the notion that CK testing should be abandoned in the era of routine hsTnT testing. Including all patients who presented to an ED of a major urban tertiary care center represents a real-world population, which allows our results to be highly applicable. This work is unique in that it included patients with baseline elevated hsTnT values (such as those with chronic kidney disease or congestive heart failure), a population in which the clinical utility of CK for diagnosis of AMI has not been studied. This allowed us to additionally assess the utility of CK testing in these patients, and thus to conclude that CK does not offer any incremental benefit to hsTnT alone.

The study center, consistent with many centers regionally and abroad, uses unfractionated CK rather than CK-MB to screen for AMI in the ED population. This is because, although the specificity for cardiac injury of CK-MB is higher than CK, the sensitivity of CK has been shown to be slightly higher than that of CK-MB [10]. Previous studies in the era of hsTnT have studied only CK-MB or CK-MB index [2, 4, 5], and this study therefore adds to the literature by showing that even screening with unfractionated CK is of no added value in diagnosis of ACS. Given the similarities in sensitivity and NPV, and findings consistent with previous work [2, 4], these results are likely applicable to CK-MB as well.

With a CK cost of approximately $4 per test at our institution, we estimate an annual cost-savings of at least $32,000 at our center alone and greater than $100,000 across the region if routine CK testing was to be eliminated for the evaluation of chest pain and diagnosis of AMI in the ED. Our results show that CK does not provide any benefit to patient care in the context of diagnosing AMI, and it is therefore an unnecessary and wasteful expenditure. In resource-limited practice environments with mounting pressure to control health-care expenditures, continued routine use of CK testing should be considered inappropriate. This cost analysis assumes one CK measurement per presentation, which is an underestimation of the real-world cost; the majority of patients had multiple serial biomarker measurements.

This study was conducted at a single center and was retrospective in nature, with all the limitations appertaining thereto. For pragmatic reasons, baseline data including prevalence of comorbidities such as CKD and CHF, which may influence hsTnT values, was not collected. However, the prevalence of CKD in the regional population has been previously reported to be 10.6% [11]. The prevalence of ischemic heart disease in this population has been reported as 6.6%, and chronic heart failure 3.8% [12]. The yearly incidence of AMI in this population has been reported as 2.1% [12]. These values are higher than the Canadian average [11, 12].

In our assessment of 30-day MACE in patients with negative hsTnT and positive CK, it is possible that our review did not identify all of the patients with a MACE. However, while we cannot say with certainty that no cases were missed, in the region where the study was conducted all regional emergency department presentations are visible in the electronic chart, and all STE-ACS, NSTE-ACS, and surgical revascularization cases are urgently referred to this cardiac center and would have been included in our analysis. It is therefore highly unlikely that MACE events would have been missed.

By defining a diagnostic hsTnT rise as ≥5 ng/L, it is possible that some patients who might have been considered diagnostic based upon this criterion may have been clinically non-diagnostic using 3 or 6 h protocols with a higher cut-off for hsTnT positivity [10], and therefore should have been included in our group of patients with non-diagnostic hsTnT and positive CK. However, we believe the effect of this limitation on our data to be minimal, as the ≥5 ng/L cut-off used is highly sensitive and specific [9], is consistent with accepted diagnostic standards for AMI [8], and is not markedly different from the ≥7 ng/L or ≥ 9 ng/L cut-offs described in the longer protocols [13]. In addition, a ≥ 5 ng/L cut-off would have prompted further serial testing and probable cardiologic consultation based upon the practice patterns of the study institution. We therefore feel it is unlikely that we would have missed a significant number of patients who would have been considered to have diagnostic hsTnT by our definition, and not to have been considered as such clinically. The generalizability of this study is limited in centers that have already eliminated CK testing; however, there exist many centers across North America that measure CK routinely [1]. It is important to note that our study did not attempt to calculate the sensitivity and specificity of these biomarkers, which has been well described elsewhere [7].

Strengths of this study include the large sample size and that the patient population studied had a high prevalence of cardiac disease, and therefore a high pre-test probability of AMI. In addition, the population studied was a real-world sample of all patients presenting to the ED of an urban tertiary center in a region with a high prevalence of CKD, CHF, and ischemic heart disease. Even in such a population, CK was not found to be useful. Building upon previous studies, our work helps define the role of CK in patients with elevated baseline hsTnT. These patients were excluded from previous studies despite making up a large proportion of patients with coronary artery disease, further underscoring the significance of our results.

## Conclusion

CK does not add value to clinical decision making regarding the diagnosis of acute myocardial infarction in the emergency department. Routine CK testing does not provide benefit to patient care and therefore represents an unnecessary system cost. Ongoing quality improvement initiatives are required to eliminate the routine use of CK and other unnecessary tests, thereby optimizing cost-effective delivery of patient care.

## Data Availability

De-identified data available from corresponding author upon reasonable request.

## Abbreviations

ACS: Acute coronary syndrome
AMI: Acute myocardial infarction
CEDIS: Canadian Emergency Department Information System
CHF: Congestive heart failure
CK: Creatine kinase
CKD: Chronic kidney disease
CK-MB: Creatine kinase myocardial band
ED: Emergency department
hsTnT: High-sensitivity Troponin T
MACE: Major adverse cardiac event
ng/L: Nanograms/liter
NPV: Negative predictive value
NSTE-ACS: Non-ST-elevation acute coronary syndrome
STE-ACS: ST-elevation acute coronary syndrome
U/L: Units/liter
